# How Aging Affects T Lymphocyte-Mediated Immunity

**DOI:** 10.3389/fimmu.2013.00296

**Published:** 2013-09-24

**Authors:** Dietmar Herndler-Brandstetter

**Affiliations:** ^1^Department of Immunobiology, Yale University School of Medicine, New Haven, CT, USA

**Keywords:** aging, biomarkers, memory T lymphocytes, lifespan, autoimmunity, vaccination, cytomegalovirus

Increasing age has been associated with an insufficient protection following vaccination and an increased incidence and severity of infectious diseases. The predicted acceleration of global population aging will accentuate the need to understand the mechanisms that drive the age-related decline of the immune system and to, eventually, identify strategies to improve immune function in elderly people. One type of immune cell appears to be most dramatically affected by the aging process: T lymphocytes. As T lymphocytes are key players of the adaptive immune system, this research topic summarizes our current understanding on how aging affects the microenvironmental niches and molecular networks that are important for the generation, survival and function of naïve, memory and effector T lymphocytes.

Age-related changes of the bone marrow and the thymus microenvironment lead to a decreased thymic output of functional, naïve T lymphocytes. The article by Palmer (1) suggests that the thymic stroma is a key factor in regulating thymic atrophy and that both, intrinsic and extrinsic factors contribute to the age-related decline in thymopoiesis. Several studies also demonstrate that the kinetics of the involution of the thymus is not uniform throughout life but can be divided into distinct phases, which are probably controlled by different molecular mechanisms.

The article by Chen et al. (2) provides an extensive review about how aging affects genome-wide transcriptional changes in different cell types, such as thymocytes, CD4 and CD8 T cell subsets in mice and humans. Gene networks and signaling pathways that are altered in aged T cells are highlighted and they may prove useful to select robust biomarkers of T cell aging. Moro-Garcia et al. (3) address the impact of aging on the phenotype and function of naïve, memory, and highly differentiated CD4^+^ T cells. The authors also propose that the homeostatic cytokine interleukin (IL)-15 can rescue functional properties of highly differentiated CD4^+^CD28^−^ T cells that accumulate in elderly people.

The impact of aging on a clinically highly relevant T cell subset, namely regulatory T cells (Treg), is reviewed by Fessler et al. (4). The authors discuss how aging affects Treg cell generation, homeostasis, diversity, and function. The review article also highlights the need to address the gaps in our understanding of Treg cell aging. This will be of relevance when it comes to implementing adoptive Treg cell therapies to treat autoimmune diseases in older individuals. The impact of aging on Treg cells is also addressed by an original article by Raynor et al. (5). The authors show that serum IL-2 levels decline during aging, which is associated with the accumulation of a unique CD25^lo^ Bim^lo^ FoxP3^+^ Treg population in old mice. Although the mechanism of the age-dependent decline in Bim expression in Treg cells remains to be determined, the accumulation of CD25^lo^ Bim^lo^ Treg cells in old mice seems to be mediated by IL-15.

The article by Goronzy et al. (6) addresses the paradox of an age-dependent decline in immune responses while autoimmune diseases increase in incidence. The authors hypothesize that the same defects that account for the decreased ability to generate protective immune responses also contribute to the increased risk of autoimmunity. The major risk factor for autoimmunity in old age appears to be the reshaping of the peripheral T cell receptor repertoire, which is driven by the age-dependent involution of the thymus. The consequent increase in homeostatic T cell proliferation prolongs peripheral T cell survival and selects for self antigen and cytokine responsiveness, thereby facilitating the generation of autoreactive T cells. Fulop et al. (7) review longitudinal studies that analyzed immune parameters predictive of survival. These studies defined an immune risk profile and showed that persistent viral infections, in particular *Cytomegalovirus* infection, can accelerate human T cell aging.

In an opinion article, Aspinall and Lang (8) focus on the impact of influenza virus infection in the elderly and highlight the current problems of designing a more effective vaccine for people older than 60 years of age. The authors propose a portfolio approach and emphasize that future developments should move toward a more personalized treatment regimen that may even be combined with therapeutic approaches that target immune aging itself. In another opinion article, Flavell and colleagues (9) accentuate the need to define a robust set of markers of T cell aging. The authors summarize our current knowledge about markers of T cell aging and emphasize the lack of data about T cell phenotypes and functions in various human organs and tissues, which would be important to better understand the fate of different T cell types and the role of specific markers. Eventually, the identification of a set of markers for immunosenescence would be a valuable clinical tool and would allow to evaluate potential therapeutic interventions aiming to prevent or reverse immune cell aging, and to personalize vaccination strategies for elderly people at risk, thereby increasing life and health span.
